# Allostatic Load Predicts Symptom Burden Among Breast Cancer Survivors

**DOI:** 10.21203/rs.3.rs-9521147/v1

**Published:** 2026-05-14

**Authors:** Yufan Guan, Jie Shen, Joseph Boyle, Katharine E. Daniel, Philip I-Fon Chow, Bernard F. Fuemmeler, Hua Zhao

**Affiliations:** University of Virginia; University of Virginia; Virginia Commonwealth University; University of Virginia; University of Virginia; Virginia Commonwealth University; University of Virginia

## Abstract

Breast cancer survivors frequently experience persistent and co-occurring symptoms, yet the role of allostatic load (AL), a measure of cumulative physiological stress, in shaping these outcomes remains unclear. We examined the associations of AL with physical and mental health outcomes, symptom burden profiles, and socioeconomic disparities among breast cancer survivors using data from the UK Biobank. The study included 1,444 breast cancer survivors and 1,444 age-matched women without cancer who completed baseline (2006–2010) and follow-up (2022) assessments. AL was derived from 11 biomarkers and analyzed as a continuous measure. Survivorship outcomes included sleep quality, fatigue, depression, anxiety, cognitive function, functional limitation, and self-rated mental health. Latent class analysis was used to identify symptom burden profiles, and multivariable regression and mediation analyses were performed. Among breast cancer survivors (mean age 58 years), higher AL was associated with poorer sleep quality (β = 0.18, 95% CI 0.06–0.29), greater fatigue (β = 0.23, 95% CI 0.12–0.35), increased functional limitation (β = 0.28, 95% CI 0.09–0.47), higher depressive symptoms (β = 0.15, 95% CI 0.02–0.28), higher anxiety (β = 0.12, 95% CI 0.01–0.23), and poorer self-rated mental health (β = −0.09, 95% CI - 0.13 to - 0.05), but not cognitive function. These associations were weaker or absent among women without cancer. Higher AL was also associated with increased likelihood of adverse symptom burden profiles. Mediation analyses indicated that AL partially mediated associations of income and education with multiple outcomes, accounting for approximately 6–15% and 8–9% of these associations, respectively. These findings suggest that elevated AL is associated with worse survivorship outcomes and contributes to socioeconomic disparities in symptom burden among breast cancer survivors. Chronic physiological stress may represent an important mechanism underlying survivorship heterogeneity and a potential target for intervention.

## Introduction

The rates of breast cancer survivorship have risen substantially in recent years, contributing to an increasing number of long-term survivors. Many of these survivors report persistent symptoms that affect their well-being, including poor sleep quality, fatigue, depression, anxiety, cognitive impairment, and functional difficulties (1-3). However, survivors who share similar tumor biology and treatment regimens differ substantially in how these symptoms present (4). This suggests that additional factors drive important symptom variations. Understanding what explains these variations is key to identifying survivors most at risk for adverse long-term outcomes and informing interventions to mitigate their development.

One of the proposed mechanisms to explain these symptom variations is chronic physiological stress, as represented by allostatic load (AL) (5, 6). AL refers to the cumulative effect of stress on the biological systems in the body, including inflammatory, metabolic, neuroendocrine, and cardiovascular systems (5, 6). Higher AL is associated with an increased risk of various illnesses, poor functional health, and mortality in unselected samples (7-9). Higher AL is also associated with reduced survival rates among breast cancer survivors (10-12). However, there is very little information linking AL with symptom burden and functional health among breast cancer survivors.

Shared biological mechanisms between physiological stress and cancer survivorship outcomes provide a strong theoretical basis for investigating the role of AL to explain variations in breast cancer survivorship outcomes (13, 14). For example, dysregulation of HPA-axis or chronic inflammation, processes central to physiological stress, could lead to impaired sleep, negative mental state, and poor physical health (15-18). These processes could therefore result in clusters of co-occurring symptoms among cancer survivors that share a common underlying biology. Additionally, socioeconomic (SES) disadvantage affects both stress biology and cancer outcomes (19, 20). Specifically, people experiencing socio-economic disadvantage have higher levels of AL and poorer health in general (19). Thus, AL may act as a mediator that links the robust association between SES and survivorship outcomes. However, evidence on the association of AL with breast cancer survivorship outcomes remains limited.

To address this gap, we leveraged data from the UK Biobank to examine whether AL, as a marker of cumulative physiological stress, contributes to the heterogeneity of physical and mental health outcomes among breast cancer survivors. Specifically, we aimed to determine whether higher AL is associated with greater symptom burden, to identify distinct patterns of co-occurring symptoms, and to evaluate whether AL helps explain socioeconomic disparities in these adverse outcomes.

## Methods

### Data Source

This study used data from the UK Biobank, a population-based cohort of over 500,000 participants aged 40–69 years recruited across the United Kingdom between 2006 and 2010. Detailed information on study design and data collection is available through the UK Biobank resource (21). For this analysis, we identified women with a history of breast cancer who completed both baseline (2006 to 2010) and follow-up assessments (2022). Women with other cancer types were excluded to reduce potential confounding. Participants were further excluded if they had missing data on any of the eleven biomarkers used to calculate AL (described below) or on symptom outcomes. A comparison group of women without a history of cancer was selected and 1:1 age-matched to breast cancer survivors. After applying all inclusion and exclusion criteria, the final analytic sample included 1,444 breast cancer survivors and 1,444 age-matched women without cancer.

### AL Score Construction

Detailed methods for constructing AL scores are documented in our previous studies (22-24). Detailed information on the 11 factors that contribute to AL scores is shown in the **Supplement** Table 1. In brief, AL score was calculated by summing the individual risk indicators across all biomarkers, resulting in a total score ranging from 0 to 11. Higher scores reflect greater cumulative physiological dysregulation across multiple biological systems. In the analyses, AL was examined as a continuous variable, as well as a categorical variable.

### Survivorship Outcomes

Key survivorship outcomes were derived from follow-up health and mental well-being questionnaires. Sleep quality was assessed using the Pittsburgh Sleep Quality Index (PSQI; fields p30442–p30467). A global score was calculated according to standard procedures, with higher scores indicating poorer sleep quality. Fatigue was assessed using six items (p28696–p28701) capturing symptom presence, duration, and activity impact. Duration was categorized (< 2 weeks, 2–4 weeks, 4–12 weeks, > 12 weeks), and impact was classified as no limitation versus reduced, avoided, or modified activities. Item-level scores were calculated as the product of duration and impact and summed to derive an overall fatigue score. Depressive symptoms were measured using the Patient Health Questionnaire (PHQ-9; p29002–p29010), with each item scored from 0 (“not at all”) to 3 (“nearly every day”) and summed to generate a total score. Anxiety symptoms were assessed using the Generalized Anxiety Disorder scale (GAD-7; p29058–p29064), with identical scoring and summation. Cognitive function was assessed using three items measuring two domains: thinking difficulties (p28720–p28722) and communication problems (p28723–p28725). For each domain, scores were calculated by multiplying duration and impact and summed to obtain an overall cognitive function score. Functional limitations were measured using items measuring difficulty performing daily activities (p28739–p28752), with responses scored from 0 (“no difficulty”) to 4 (“extreme difficulty or unable to perform”). Scores were summed to derive a total functional limitation score. Self-rated mental health was assessed using a single item (p29155) with ordered response categories ranging from poor to excellent. This variable was treated as continuous in analyses, with higher scores indicating better mental health.

### Covariates

To control for potential confounding, models were adjusted for demographic, SES, lifestyle, and clinical factors. Demographic variables included age at recruitment and race/ethnicity. SES indicators included educational attainment (≤ vs. > high school), household income (dichotomized at £31,000), and the Townsend Deprivation Index (categorized as high vs. low based on the cohort median). Lifestyle factors included smoking status, alcohol consumption, and physical activity. Smoking status was based on self-reported tobacco use. Alcohol intake was categorized as “special occasions or never,” “moderate,” or “heavy.” Physical activity was assessed using metabolic equivalent of task (MET) scores and classified as low, moderate, or high. Baseline sleep quality was included to account for pre-existing sleep conditions.

### Latent class analysis

**(LCA)** was used to identify patterns of co-occurring symptoms across six symptom domains, including sleep disturbance, fatigue, depression, anxiety, cognitive dysfunction, and functional limitation. For each domain, clinically relevant symptoms were defined as binary indicators (present vs. absent). Competing models with varying numbers of classes were evaluated, and the optimal solution was selected based on model fit indices, interpretability, and class separation. Participants were assigned to their most likely class based on posterior probabilities. Class-specific item-response probabilities were visualized using heatmaps to characterize symptom profiles across classes.

### Statistical analysis

Descriptive statistics were used to summarize participant characteristics. Continuous variables were reported as means with standard deviations (SD), and categorical variables as counts with percentages. Differences between breast cancer survivors and participants without cancer were assessed using two-sample *t*-tests for continuous variables and chi-square tests for categorical variables. Associations between AL and symptom outcomes were evaluated using linear regression, with AL modeled as a continuous exposure. Regression coefficients and 95% confidence intervals (CIs) were estimated. Models were adjusted for age at AL measurement, race/ethnicity, education, income, Townsend score, smoking, alcohol consumption, physical activity, sleep quality, time from AL measurement to follow-up questionnaire, and among breast cancer survivors, time from cancer diagnosis to AL measurement. Then, we assessed whether AL mediated between SES factors (income and education) and survivorship outcomes. We estimated indirect effect, direct effect, and total effect using bootstrap-derived 95% CI (25). Finally, associations between AL and latent symptom patterns identified through LCA were examined using multinomial logistic regression, with the lowest symptom burden class as the reference group. AL was modeled both as a continuous variable and as a binary indicator comparing participants in the highest quartile (≥ 75th percentile) with all others. Results were reported as odds ratios (ORs) with 95% CIs. All analyses were conducted using R (version 4.2). Statistical tests were two-sided, with *P* < 0.05 considered statistically significant.

## Results

Baseline characteristics between breast cancer survivors and age-matched non-cancer women were largely comparable, including race/ethnicity, education, Townsend deprivation index, smoking status, and physical activity (all *P* > 0.05; Table 1). However, survivors were more likely to have lower household income and differed in the distribution of self-reported alcohol consumption (both *P* < 0.001), and a higher proportion reported frequent insomnia (37.7% vs. 28.1%, *P* < 0.001). During follow-up, breast cancer survivors had significantly worse outcomes across all domains (all *P* ≤ 0.006).

**Figure 1:**
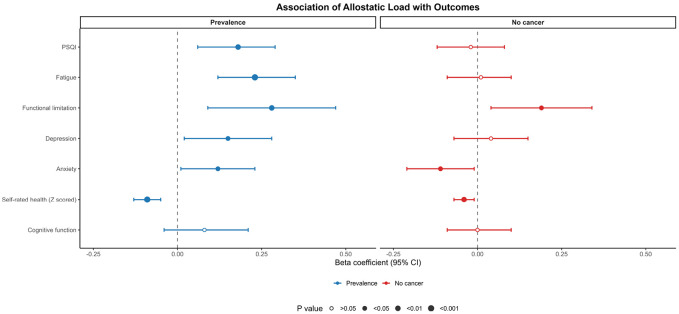
Association of AL with symptom outcomes among breast cancer survivors. Forest plots show regression coefficients and 95% confidence intervals (CIs) for the association between allostatic load and symptom outcomes in the prevalence(blue) and women without cancer (red). Points represent estimated coefficients and horizontal lines indicate 95% CIs. Outcomes include sleep quality (PSQI), fatigue, depression, anxiety, functional limitation, and self-rated mental health (Z-score). Circle size reflects the level of statistical significance (p-value), with larger circles indicating smaller p-values, while empty circles indicate non-significant associations (p ≥ 0.05)

As shown in Fig. 1 and **Supplement** Table 2, higher baseline AL was consistently associated with worse survivorship outcomes among breast cancer survivors. Each one-unit increase in AL was associated with poorer sleep quality (PSQI: β = 0.18, 95% CI: 0.06–0.29; *P* = 0.003), greater fatigue (β = 0.23, 95% CI: 0.12–0.35; *P* < 0.001), increased functional limitation (β = 0.28, 95% CI: 0.09–0.47; *P* = 0.003), higher depressive symptoms (PHQ-9: β = 0.15, 95% CI: 0.02–0.28; *P* = 0.029), and greater anxiety (GAD-7: β = 0.12, 95% CI: 0.01–0.23; *P* = 0.036). Higher AL was also associated with poorer self-rated mental health (β = − 0.09, 95% CI: −0.13 to - 0.05; *P* < 0.001), while no significant association was observed for cognitive function. In contrast, among women without cancer, significant associations were observed only for functional limitation (β = 0.19, 95% CI: 0.04–0.34; *P* = 0.011) and self-rated mental health (β = − 0.04, 95% CI: −0.07 to − 0.01; *P* = 0.007), and an inverse association was observed for anxiety (β = [SIP]- 0.11, 95% CI: −0.21 to − 0.01; *P* = 0.036).

Mediation analyses indicated that AL significantly mediated the associations between income and education with several symptom outcomes (Table 2). For income, AL mediated the associations with sleep quality (indirect effect β = −0.06, 95% CI: −0.12 to − 0.01; total β = −0.69, 95% CI: −1.09 to − 0.30), fatigue (indirect effect β = −0.07, 95% CI: −0.13 to [SIP]- 0.02; total β = −0.46, 95% CI: −0.86 to − 0.06), depression (indirect effect β = −0.05, 95% CI: −0.11 to − 0.01; total β = −0.61, 95% CI: −1.06 to − 0.15), functional limitation (indirect effect β= −0.10, 95% CI: −0.20 to − 0.03; total β = −1.66, 95% CI: −2.35 to − 1.06), and self-rated mental health (indirect effect β = 0.03, 95% CI: 0.01–0.05; total β = 0.23, 95% CI: 0.09–0.37). These indirect effects accounted for approximately 6–15% of the total associations.

Similarly, AL significantly mediated the associations between education and sleep quality (indirect effect β = −0.06, 95% CI: −0.11 to − 0.02; total β = −0.68, 95% CI: −1.06 to − 0.33) and functional limitation (indirect effect β = −0.10, 95% CI: −0.19 to − 0.03; total β = −1.09, 95% CI: −1.65 to − 0.48), with AL explaining approximately 8–9% of the total association.

**Figure 2:**
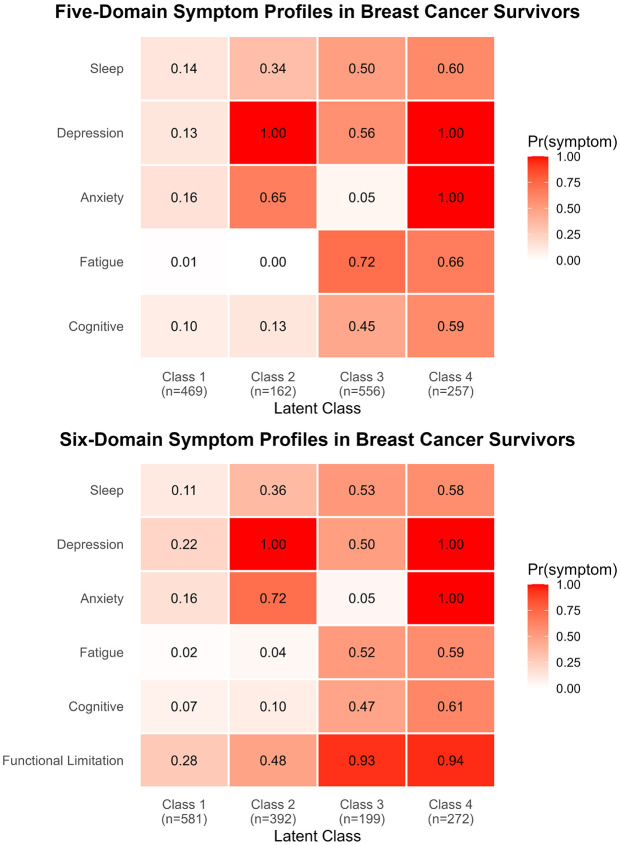
Latent symptom profiles among breast cancer survivors. Heatmaps show the probability of symptom endorsement across latent classes identified using latent class analysis. The six-domain model included sleep disturbance, depression, anxiety, fatigue, cognitive symptoms, and functional limitation. Colors indicate the probability of symptom presence within each class, with darker shading representing higher probability. Classes are ordered from Class 1 (lowest symptom burden) to Class 4 (highest symptom burden), and the number of participants in each class is shown on the x-axis.

Latent class analysis identified four distinct symptom profiles in six-domain model, representing a gradient from low to severe symptom burden (Fig. 2 and **Supplementary Table 3**). Model fit statistics supported the four-class solution as optimal; it improved fit over three-class models (Class 4: AIC = 9686.30, BIC = 9828.73) without meaningful gains in interpretability from additional classes. In the six-domain model, classes were: low symptom (Class 1, n = 548), intermediate burden (Class 2: n = 463), functionally impaired (high limitation with moderate symptoms) (class 3: n = 192), and severe multi-domain burden (Class 4: n = 241).

Finally, we assessed relationships between AL and latent class memberships (Table 3). Each one-unit increase in AL was associated with higher odds of belonging to more severe symptom classes (Class 3: OR = 1.16, 95% CI: 1.04–1.30; Class 4: OR = 1.18, 95% CI: 1.06–1.31). In further categorical analysis, participants with high AL (≥ 75th percentile) had markedly higher odds of belonging to the most adverse symptom classes (Class 3: OR = 1.52, 95% CI: 1.44–1.61; Class 4: OR = 1.99, 95% CI: 1.73–2.28).

## Discussion

In this large population-based study, we found that higher baseline AL was consistently associated with worse survivorship outcomes among breast cancer survivors, including poorer sleep quality, greater fatigue, worse psychological well-being, and increased functional limitation. These associations were substantially stronger among breast cancer survivors than age-matched non-cancer women. In addition, higher AL was associated with distinct patterns of symptom burden, as individuals with elevated AL were more likely to belong to adverse, multi-symptom profiles identified through latent class analysis. Mediation analyses further indicated that AL partially mediated the relationships between SES (income and education) and multiple symptom outcomes, suggesting that cumulative physiological stress represents a key pathway linking social disadvantage to survivorship burden.

While previous studies have linked higher AL to increased breast cancer mortality (10-12), its impact on symptom and functional health has been less well understood. In this study, higher AL was consistently associated with worse symptoms, functional limitation, and poorer mental well-being, suggesting that AL reflects not only long-term health risk but also worse day-to-day functioning among breast cancer survivors. The stronger associations observed among breast cancer survivors compared with non-cancer women suggest that AL captures a heightened state of physiological vulnerability in the posttreatment period (26). This likely reflects the combined effects of treatment-related physiological dysregulation, reduced biological reserve, and ongoing psychosocial stressors, which together amplify susceptibility to the downstream effects of cumulative stress (27, 28).

These findings align with a growing but still limited body of literature indicating that higher AL is associated with poorer health-related quality of life, greater depressive symptoms, and increased functional impairment across cancer populations (29-31). Prior studies in lung and breast cancer have shown that lower AL is linked to delayed deterioration in quality of life and symptom burden, whereas higher AL is associated with worse functional well-being (29-31). However, existing studies are limited in number, often heterogeneous and cross-sectional in design, and have primarily focused on depressive symptoms and quality of life, with other survivorship outcomes less well studied. Taken together, current evidence supports an emerging role for AL in cancer survivorship and underscores the need for longitudinal studies to clarify its clinical relevance.

The observed associations are biologically plausible and likely reflect shared underlying mechanisms of stress-related physiological dysregulation (13, 14). Chronic activation of stress-response systems, particularly the HPA axis and autonomic nervous system, can alter inflammation, circadian rhythms, and neuroendocrine signaling (14, 32-34). In the context of cancer and its treatment, these processes may further interact with tumor–brain signaling pathways, leading to disruption of hypothalamic and limbic circuits that regulate sleep, energy, mood, and cognition (32, 34, 35). As a result, survivors may experience a cluster of interrelated symptoms, including insomnia, persistent fatigue, depression, anxiety, and cognitive impairment. These processes likely reinforce one another over time, creating a feedback loop in which physiological dysregulation and symptom burden co-evolve. Our findings, particularly the strong associations between AL and sleep, fatigue, and functional limitation, are consistent with this multisystem, interconnected framework.

Importantly, our latent class analysis demonstrated that survivorship symptoms cluster into distinct profiles rather than occur independently, which is consistent with previous reports (1, 3, 36). Across six-symptoms model, we identified a gradient of symptom burden, including low, intermediate, and high multi-symptom groups, as well as profiles characterized by psychological or functional dominance. Higher AL was associated with increased likelihood of membership in the most adverse classes, including those characterized by severe, multi-domain symptom burden. These findings suggest that AL may play a central role in shaping not only the severity but also the patterning of survivorship symptoms, supporting the concept of a shared biological substrate underlying symptom cluster.

Another notable finding is the heterogeneity of associations by SES, alongside evidence for mediation. The effects of AL on multiple outcomes were consistently stronger among individuals with lower education and income, suggesting that socioeconomic disadvantage may amplify the impact of physiological stress on survivorship (**Supplement Table 4**). In addition, mediation analyses indicated that AL partially mediated the associations between income and education with several symptom outcomes, supporting a pathway in which social disadvantage contributes to elevated physiological stress, which in turn shapes survivorship burden. These findings are consistent with prior studies showing that social adversity is linked to higher AL and worse health outcomes (19, 20), and align with the report by Petrova et al., in which the association between AL and depressive symptoms was more pronounced among individuals with lower SES (29).

This study has several important strengths. We leveraged a large, well-characterized population-based cohort with detailed information on both biological and patient-reported outcomes. The use of an age-matched non-cancer comparison group allowed us to contextualize findings and demonstrate that the impact of AL is amplified in cancer survivorship. The integration of multiple outcomes and the use of latent class analysis provided a comprehensive assessment of symptom burden and its underlying structure. In addition, the examination of SES modifiers offers important insights into disparities in survivorship.

Several limitations should also be considered. First, AL was assessed at a single time point, which may not fully capture dynamic changes in physiological stress over time. Second, the UK Biobank lacks detailed clinical and treatment information. Key variables such as tumor stage, treatment regimens, treatment duration, and toxicity profiles are not comprehensively captured, which may limit the ability to fully account for disease severity and treatment-related effects in the analyses. Third, survivorship outcomes were based on self-reported measures, which may be subject to measurement error. Finally, the UK Biobank population may not be fully representative (37, 38), which could limit generalizability.

In conclusion, higher AL is strongly associated with worse survivorship outcomes and greater symptom burden among breast cancer survivors, with effects more pronounced than in women without cancer. Mediation analyses further suggest that AL partially explains the link between SES and symptom outcomes, highlighting chronic physiological stress as a key pathway underlying survivorship disparities. These findings underscore the role of stress biology in shaping survivorship heterogeneity and suggest that targeting AL may offer opportunities to improve long-term outcomes. Future studies should examine the dynamic interplay between AL and symptom trajectories and evaluate interventions aimed at reducing physiological stress to enhance survivorship.

## Supplementary Material

This is a list of supplementary files associated with this preprint. Click to download.

• SupplementTable1.docx

## Figures and Tables

**Table 1:** Description of baseline characteristics and symptoms during the follow-up among breast cancer survivors and age matched non-cancer women

Variable	Breast cancer survivors (N = 1,444)	Non-cancer women (N = 1,444)	P value
**Baseline characteristics**				
Age at enrollment, Mean (SD)	57.99 (6.39)	57.99 (6.39)	> 0.999
Race/ethnicity, N (%)			0.202
White	1,418 (98.40%)	1,411 (97.92%)	
Asian	7 (0.49%)	11 (0.76%)	
Black	1 (0.07%)	6 (0.42%)	
Mix or others	15 (1.04%)	13 (0.90%)	
Education, N (%)			0.602
>High school	710 (53.06%)	718 (54.15%)	
≤High school	628 (46.94%)	608 (45.85%)	
Income level, N (%)			< 0.001
≤£30,999	558 (44.60%)	484 (37.58%)	
>£30,999	693 (55.40%)	804 (62.42%)	
Townsend score (by median)			0.711
High	634 (43.97%)	624 (43.21%)	
Low	808 (56.03%)	820 (56.79%)	
Cigarette smoking status, N (%)			0.984
Never	820 (56.90%)	817 (56.66%)	
Former	539 (37.40%)	541 (37.52%)	
Current	82 (5.69%)	84 (5.83%)	
Alcohol drinking status, N (%)			< 0.001
Special occasions or never	269 (18.63%)	192 (13.30%)	
Moderate	527 (36.50%)	492 (34.07%)	
Heavy	648 (44.88%)	760 (52.63%)	
Physical activity, N (%)			0.890
Low	229 (18.63%)	224 (18.02%)	
Moderate	544 (44.26%)	548 (44.09%)	
High	456 (37.10%)	471 (37.89%)	
Sleep quality (Insomnia), N (%)			< 0.001
Never/rarely	230 (15.93%)	379 (26.25%)	
Sometimes	669 (46.33%)	660 (45.71%)	
Usually	545 (37.74%)	405 (28.05%)	
**Symptom during follow-up**			
PSQI, Mean (SD)	7.16 (3.51)	6.50 (3.41)	< 0.001
Fatigue, Mean (SD)	2.28 (3.26)	1.80 (2.88)	< 0.001
Depression (PHQ9), Mean (SD)	2.51 (3.52)	2.08 (3.16)	< 0.001
Anxiety (GAD7), Mean (SD)	1.85 (2.90)	1.53 (2.83)	0.003
Cognitive function, Mean (SD)	1.57 (3.48)	1.24 (2.92)	0.006
Functional limitation, Mean (SD)	3.87 (5.41)	3.13 (4.73)	< 0.001
Self-rated health (Z scored), Mean (SD)	−0.09 (1.09)	0.09 (0.92)	< 0.001

**Table 2:** AL mediates of the association between SES factors and survivorship outcomes among breast cancer survivors

Outcome	Family income (>£30,999 vs ≤£30,999)							
	Indirect effect β (95% CI)	P value	Direct effect β (95% CI)	P value	Total effect β (95% CI)	P value	Mediated (%)
PSQI	−0.06 (−0.12, −0.01)	0.008	−0.64 (−1.03, −0.24)	0.004	−0.69 (−1.09, −0.30)	0.002	8
Fatigue	−0.07 (−0.13, −0.02)	0.002	−0.39 (−0.78, 0.02)	0.056	−0.46 (−0.86, −0.06)	0.010	15
Depression	−0.05 (−0.11, −0.01)	0.012	−0.56 (−1.01, −0.09)	0.022	−0.61 (−1.06, −0.15)	0.010	8
Anxiety	−0.03 (−0.08, −0.00)	0.042	−0.24 (−0.63, 0.13)	0.224	−0.27 (−0.67, 0.10)	0.150	9
Cognitive function	−0.03 (−0.08, 0.00)	0.084	−0.04 (−0.43, 0.36)	0.858	−0.06 (−0.47, 0.33)	0.772	6
Functional limitation	−0.10 (−0.20, −0.03)	0.006	−1.57 (−2.23, −0.96)	< 0.001	−1.66 (−2.35, −1.06)	< 0.001	6
Self-rated mental health	0.03 (0.01, 0.05)	0.008	0.20 (0.07, 0.34)	0.006	0.23 (0.09, 0.37)	0.002	12
	Education attainment (> High school vs ≤High school)						
PSQI	−0.06 (−0.11, −0.02)	< 0.001	−0.63 (−1.02, −0.25)	0.002	−0.68 (−1.06, −0.33)	< 0.001	8
Fatigue	−0.07 (−0.13, −0.03)	< 0.001	−0.15 (−0.50, 0.20)	0.408	−0.22 (−0.57, 0.14)	0.238	25
Depression	−0.05 (−0.10, −0.01)	0.012	−0.26 (−0.67, 0.12)	0.212	−0.31 (−0.71, 0.07)	0.124	13
Anxiety	−0.03 (−0.07, 0.00)	0.062	−0.29 (−0.65, 0.03)	0.080	−0.32 (−0.66, −0.00)	0.044	8
Cognitive function	−0.02 (−0.06, 0.01)	0.170	−0.41 (−0.80, −0.05)	0.034	−0.44 (−0.82, −0.07)	0.028	5
Functional limitation	−0.10 (−0.19, −0.03)	< 0.001	−0.99 (−1.57, −0.37)	< 0.001	−1.09 (−1.65, −0.48)	< 0.001	9
Self-rated mental health	0.03 (0.01, 0.05)	0.004	−0.01 (−0.14, 0.11)	0.838	0.01 (−0.12, 0.13)	0.818	26

*. All models were adjusted for age, race, time from baseline AL measurement to questionnaires, and time from baseline AL measurement to cancer diagnosis.

**Table 3:** Associations between AL and latent classes among breast cancer survivors

Class 1	AL (continuous)	AL (≥ 75% vs < 75%)
	Reference	Reference
Class 2	1.03 (0.94, 1.13)	1.18 (0.95, 1.46)
Class 3	1.16 (1.04, 1.30)	1.52 (1.44, 1.61)
Class 4	1.18 (1.06, 1.31)	1.99 (1.73, 2.28)

*. Adjusted by age of AL measurement, race, education, income, Townsend score, smoking status, alcohol drinking status, physical activity, sleep quality, time from AL measurement to follow-up questionnaires, and time from cancer diagnosis to AL measurement.

## Data Availability

Data available: No Data underlying this study were accessed through the UK Biobank under application number 94449. Researchers who meet UK Biobank eligibility criteria may obtain access to the same data by submitting an independent application via the UK Biobank Access Management System (https://www.ukbiobank.ac.uk/enable-your-research/apply-for-access).
